# Die Relevanz von Netzwerkarbeit in der schulischen Gesundheitsförderung

**DOI:** 10.1007/s00103-022-03553-8

**Published:** 2022-06-13

**Authors:** Jan Josupeit, Kevin Dadaczynski, Eike Quilling

**Affiliations:** 1 Department für Angewandte Gesundheitswissenschaften, Hochschule für Gesundheit Bochum, University of Applied Sciences, Gesundheitscampus 6–8, 44801 Bochum, Deutschland; 2 Fachbereich Gesundheitswissenschaften, Hochschule Fulda, Fulda, Deutschland

**Keywords:** Lebenswelt, Förderfaktoren, Erziehungsauftrag, Handlungsstrategien, Lifeworld, Supportive factors, Educational mandate, Action strategies

## Abstract

Die Schule ist einer der wichtigsten Orte, an dem die Förderung der Gesundheit von Kindern und Jugendlichen ansetzen kann, u. a. weil die Schülerinnen und Schüler hier einen Großteil ihrer Zeit verbringen. Allerdings kann sich Schule nur in Teilen der Gesundheitsförderung widmen und die Schule ist selbst nur ein Teil der alltäglichen Umwelt, von der das Gesundheitsverhalten geprägt wird. Um die Effektivität der Gesundheitsförderung zu steigern, erscheint die Bildung von Netzwerken zwischen schulischen und kommunalen Akteurinnen und Akteuren wie Sportvereinen, Jugendhilfe, Beratungsstellen und Gesundheitsämtern sinnvoll.

Der vorliegende Artikel thematisiert die Frage der Relevanz von Netzwerken im Kontext der schulischen Gesundheitsförderung. Die Herleitung basiert einerseits auf dem gesetzlichen Rahmen des Bildungsauftrags von Schulen und dem sogenannten Präventionsgesetz von 2015 und andererseits auf der Ottawa-Charta der Weltgesundheitsorganisation (WHO) sowie auf dem daraus entwickelten Diskurs um die Begriffe „Gesundheitsförderung“ und „Settingansatz“. Es werden Perspektiven aufgezeigt, wie Netzwerkarbeit wissenschaftlich fundiert gestaltet werden kann und wie passende Netzwerkpartner gewonnen werden können. Mögliche Risiken und Chancen der Netzwerkarbeit werden analysiert und Faktoren des Gelingens und Scheiterns aufgezeigt.

Netzwerkarbeit sollte für Schulen im Sinne der Gesundheitsförderung obligatorisch sein. Sie kann dazu beitragen, Bedarfe innerhalb der Schule zu ermitteln, und gleichzeitig ein Schlüssel im Umgang mit resultierenden Herausforderungen sein. Netzwerkarbeit erfordert vor allem die Motivation der Akteurinnen und Akteure. Eine gemeinsame Vision, feste Strukturen und Kontinuität sowie entsprechende personelle Überlegungen tragen zum Gelingen der Zusammenarbeit bei.

## Abstract (en)

School is one of the most important places to start promoting the health of children and young people, partly because pupils spend a large part of their time here. However, school can only be partially dedicated to health promotion, and school itself is only part of the everyday environment by which health behaviour is shaped. In order to increase the effectiveness of health promotion, the formation of networks between school and municipal actors such as sports clubs, youth welfare services, counselling centres and health authorities seems to make sense.

This article addresses the question of the relevance of networks in the context of school health promotion. The derivation is on the one hand based on the legal framework of the educational mandate of schools and the so-called Prevention Act of 2015 and on the other hand on the Ottawa Charter of the World Health Organization (WHO) as well as on the discourse developed from it around the terms “health promotion” and “setting approach”. Perspectives are shown on how networks can be designed in a scientifically sound way and how suitable network partners can be won. Possible risks and opportunities of networking are analysed, and factors of success and failure are pointed out.

Networking should be obligatory for schools in terms of health promotion. It can help identify needs within the school and at the same time be a key in dealing with resulting challenges. Networking requires above all the motivation of the actors. A common vision, fixed structures, continuity and appropriate personnel considerations contribute to the success of the cooperation.

## Einleitung

Vor dem Hintergrund, dass Gesundheitsförderung kein abgestecktes Feld bzw. keinen festen Ort der Vermittlung hat und Teil des (pädagogischen) Handelns verschiedener Fachkräfte und Institutionen ist, ergeben sich automatisch Berührungspunkte zu unterschiedlichen Professionen. Gleichzeitig werden Professionen und Institutionen Teil der Gesundheitsförderung, deren primäre Aufgabe (und Ausbildung) nicht auf die Förderung von Gesundheit zielt. Gesundheitsförderung ist folglich Teil des Handelns verschiedener Professionen und findet felderübergreifend statt [1].

Neben dem Zuhause ist die Schule einer der wichtigsten Orte der Gesundheitsförderung in Bezug auf Kinder und Jugendliche, weil dort durch die Schulpflicht nahezu alle Kinder erreicht werden können und somit das in der Gesundheitsförderung oft gegebene Problem, bestimmte (vulnerable) Gruppen zu erreichen, hier, zumindest in Bezug auf die Anwesenheit der Adressatinnen und Adressaten, deutlich geringer ausfällt. Zudem verbringen Kinder und Jugendliche einen Großteil ihrer Zeit in der Schule.

Gleichzeitig hat die Schule einen im jeweiligen Schulgesetz definierten Bildungsauftrag, der nur zu Teilen explizit auf die Förderung der Gesundheit der Schülerinnen und Schüler zielt [2]. Entsprechend der in der Ottawa-Charta der Weltgesundheitsorganisation (WHO) formulierten These, dass „Gesundheit … von Menschen in ihrer alltäglichen Umwelt geschaffen und gelebt [wird]: dort wo sie spielen, lernen, arbeiten und lieben“ [3], stellt Schule nur einen Ausschnitt der angesprochenen alltäglichen Umwelt von Kindern und Jugendlichen dar. Daraus hervorgehend werden in der Ottawa-Charta [3] 3 Handlungsstrategien zur Gesundheitsförderung formuliert: (1) die Interessenvertretung, (2) das Ermöglichen und Befähigen sowie (3) die Vermittlung und Vernetzung [4].

Zwar erreicht Schule nahezu alle Kinder und Jugendlichen, jedoch bilden Faktoren, wie zum Beispiel Curriculum, Personal und räumliche Ausstattung, einen Rahmen, der im Sinne der Gesundheitsförderung hinsichtlich der Gestaltung von Aktivitäten, der zeitlichen Ressourcen und der Mitwirkung der Schülerinnen und Schüler einen anderen (teilweise geringeren) Handlungsspielraum eröffnet. Sportvereine, die offene Kinder- und Jugendarbeit oder die Kommune haben hier beispielsweise mehr bzw. andere Gestaltungsspielräume. Gleichzeitig fehlt diesen Institutionen an manchen Stellen insofern der Zugang zu Schülerinnen und Schülern, als sie aktiv mit ihnen in Kontakt treten müssen.

Vor diesem Hintergrund erfordert eine effektive schulische Gesundheitsförderung Netzwerkarbeit der schulischen und im weiteren Sinne kommunalen Akteurinnen und Akteure. Ziel dieses Beitrags ist die theoretische Herleitung der Rolle von Netzwerkarbeit in Schulen. Darüber hinaus werden Schwierigkeiten sowie Potenziale und Perspektiven von Netzwerkarbeit in der schulischen Gesundheitsförderung aufgezeigt. Dazu soll zunächst ein kurzer Blick auf den Begriff des Netzwerks bzw. den Begriff der Netzwerkarbeit im Kontext von schulischer Gesundheitsförderung geworfen werden. Anschließend werden relevante Akteurinnen und Akteure benannt und anhand des Rückgriffs auf verschiedene theoretische Hintergründe sowie ausgewählte empirisch ermittelte Befunde zentrale Problemlagen und Lösungsmöglichkeiten aufgezeigt.

## Hintergrund und Ausgangslage – Gesundheitsförderung, Schule und Lebenswelten

Um die Rolle von Netzwerkarbeit von Schulen in Bezug auf Gesundheitsförderung zu beleuchten und deren Potenziale sowie Perspektiven aufzeigen zu können, wird an dieser Stelle zunächst der Auftrag der Schule näher betrachtet.

Der Bildungsauftrag der Schulen in der Bundesrepublik Deutschland ergibt sich im Sinne des Föderalismus aus den Landesgesetzgebungen, die sich an dieser Stelle teilweise (deutlich) unterscheiden. Trotzdem formulieren nahezu alle Schulgesetze die Bedeutung der Förderung der Gesundheit bzw. einer gesunden Lebensweise bereits in den grundlegenden Verfügungen^1^. Mit dem im Zuge des sogenannten Präventionsgesetzes neu gefassten § 20a Abs. 3 des Fünften Buchs Sozialgesetzbuch (SGB V) wird (für gesetzlich Krankenversicherte) die Schule als „Lebenswelt“ und damit gleichzeitig als „Ort von Gesundheitsförderung und Prävention“ definiert.

Der Auftrag für Schulen zur Gesundheitsförderung (und in Bezug auf § 20a SGB V auch für den Spitzenverband der Krankenkasse) ist somit gesetzlich zwar unterschiedlich geregelt (s. oben), aber doch deutlich.

Aus der Gesetzgebung resultieren jedoch mindestens 4 Problemstellungen, die in den weiteren theoretischen Überlegungen aufgegriffen werden müssen:

1. Der im Gesetz verwendete Lebensweltbegriff ist im Vergleich zum wissenschaftlichen Diskurs sehr eng gefasst. So definiert § 20a Abs. 1 Lebenswelten als „für die Gesundheit bedeutsame, abgrenzbare soziale Systeme insbesondere des Wohnens, des Lernens, des Studierens, der medizinischen und pflegerischen Versorgung sowie der Freizeitgestaltung einschließlich des Sports“ und verwendet damit einen systemtheoretischen und metatheoretischen Lebensweltbegriff, der im Verhältnis zur originären Diskurslinie deutlich weniger subjektorientiert und somit enger ist (vgl. z. B. [8, 9]).
2. Die Schulen verfügen zunächst, neben gesundheitsbezogenen Schulfächern wie Sport oder Kochen in der Regel über kein explizites Format zur Gesundheitsförderung [10].
3. Lehrkräften mangelt es, neben durch das Curriculum bedingt fehlenden Zeitressourcen, teilweise auch an der entsprechenden Qualifikation [11].
4. Gesundheitsförderung selbst kann sich qua Definition, also zum Beispiel im Sinne der Ressourcenaktivierung oder -stärkung [4, 10], nicht nur auf den Ort Schule beziehen, wie bereits aus der oben genannten Definition der Ottawa-Charta [3] deutlich wurde.

Abgesehen von der Ottawa-Charta [3] lässt sich der vierte Gedanke auch aus dem Kritikpunkt am ungenauen Lebensweltbegriff begründen. Das ursprüngliche Verständnis der Lebenswelt, das sich bei Husserl [12] wiederfindet, und die anknüpfenden Diskurse in der Soziologie (vgl. z. B. [9]) und Philosophie [13, 14], aus phänomenologischer wie auch kommunikationstheoretischer Perspektive, zeigen einen deutlich breiteren bzw. komplexeren Begriff. So weisen Schütz und Luckmann [9] z. B. auf die Bedeutung des Bewusstseins, der Selbsterfahrung, der Zeit(perspektive), der Sprache und viele weitere Eigenschaften bzw. Strukturen hin, die Lebenswelten bedingen und damit auch definieren. Habermas [13, 14] betont in seinem philosophisch geprägten, kommunikationstheoretischen Ansatz zudem die Bedeutung von kultureller Transmission und diskutiert die kommunikative Zugänglichkeit von Lebenswelten. Die hier nur skizzenhaft mögliche Darstellung zeigt bereits anhand der Auszüge, dass der Lebensweltbegriff in den aktuellen gesetzlichen Bestimmungen aus einer ganz anderen Perspektive betrachtet wird, indem Lebenswelten als eindimensional zweckbezogene Systeme definiert werden.

Mit Blick auf den Ort oder das soziale System (Setting) Schule lässt sich der Sinn einer breiteren Verwendung des Lebensweltbegriffs und dessen immense Bedeutung für die Frage, warum es zur Erreichung der oben genannten Zielsetzungen von Schule zwingend erforderlich ist, Gesundheitsförderung als Netzwerkarbeit zu betreiben, sehr gut illustrieren. Ausgehend von dem in der Ottawa-Charta [3] formulierten Gedanken, dass Gesundheit dort geschaffen wird, wo Menschen „spielen, lernen, arbeiten und lieben“, wird deutlich, dass entsprechend Gesundheitsförderung an nicht nur einem Ort oder auch in nur einem Setting entsteht, sondern an verschiedenen miteinander interagierenden Orten und Settings. Entsprechend findet die Idee von Gesundheit, die an mehreren Orten parallel und miteinander wechselwirkend entsteht oder geschaffen wird, bei einem synonym zum „Setting“ verwendeten Begriff der „Lebenswelt“ – so wie er als eine Interpretation auch bei Engelmann und Halkow [8] auftaucht – keine Berücksichtigung. Zudem übersieht diese Betrachtungsweise, dass unter anderem die kommunikativen Überlieferungen (Transmissionen) und Interpretationen der Umwelt, die Lebenswelten prägen (z. B. Joggen gehen, rauchen oder Fahrrad fahren), aus vielen verschiedenen und miteinander komplementär oder kollidierend im Verhältnis stehenden Eindrücken bzw. Interpretationen entstehen.

Das bedeutet einerseits, dass die gesundheitsförderliche Praxis von Bewegung, Ernährung, Stressregulation oder Hygiene (vgl. [10]) Menschen kommunikativ erreichen muss – so auch die Existenz und der Zweck von Sportvereinen oder Beratungsstellen –, sowie bedeutet es andererseits, dass das Handeln von Menschen an anderen Orten auch in gesundheitsschädigender Weise geprägt werden kann.

Um eine gesundheitsförderliche Umwelt für Kinder und Jugendliche in ihrer Lebenswelt zu schaffen, ist es erforderlich, den Auftrag entsprechend deutlich breiter zu interpretieren und die Schule als einen Ort von Gesundheitsverhalten zu sehen, der gleichzeitig in Wechselwirkung mit anderen Orten steht, an denen Gesundheitsverhalten entstehen kann. Darüber hinaus sollte die Schule auch einen Schlüssel für andere Akteurinnen und Akteure darstellen, um ihre gesundheitsförderlichen Angebote zu verbreiten. Paulus [15] begründet diesen Gedanken auch mit dem resultierenden „Impact“ von Gesundheitsförderung (in Schulen), also dem aus der Vernetzung heraus entstehenden stärkeren gesundheitsförderlichen Effekt. Entsprechend muss Schule, um diesem Anspruch gerecht zu werden, netzwerkorientiert handeln bzw. Netzwerkarbeit betreiben.

## Netzwerk, Schule und Gesundheit

Netzwerke weisen bestimmte Eigenschaften auf, mittels derer sie sich identifizieren sowie anhand derer sie sich zielgerichtet konstruieren lassen. Nach Quilling et al. [16] lassen sich dabei 4 Elemente an nahezu allen Netzwerken ausmachen: Kooperation, Emergenz, Innovation und Leitbild. *Kooperation* meint dabei zunächst Austauschprozesse zwischen den Akteurinnen und Akteuren sowie die (daraus) entstehende (autopoetische) Struktur und Kontrolle. Das letztgenannte (Neu‑)Entstehen von regulierten Strukturen, welches die Kooperation im fortlaufenden Prozess bestimmt, beschreibt der Begriff der *Emergenz*. Im Zuge der fortlaufenden Kooperation verändern sich etablierte Systeme, was die *Innovation* von Netzwerken ausmacht. Gleichzeitig folgt das Netzwerk einer Vorstellung davon, wohin sich dieses entwickeln soll. Optimalerweise ist diese Vision als *Leitbild* abgestimmt.

Die Zielsetzung der Netzwerkarbeit bzw. des Bildens von Netzwerken in Schulen ist weiter oben bereits begründet worden. Nach der Beschreibung der grundlegenden Eigenschaften von Netzwerken stellt sich nun die Frage, welchen Vorteil diese Form der gemeinschaftlichen Zusammenarbeit für Institutionen, in diesem Fall Schulen, haben kann und wie sie gelingen kann. Hierzu geben Quilling et al. [16] ebenfalls einen umfangreichen Überblick.

Netzwerke sind zunächst in der Lage, Informationsdefizite von einzelnen Akteurinnen und Akteuren zu verringern. Entsprechend könnten sich Schulen durch Netzwerke über gesundheitsförderliche Angebote in der Kommune informieren oder Hinweise in Bezug auf Finanzierung von gesundheitsfördernden Angeboten in Schulen einholen. Darüber hinaus sind durch interprofessionelle oder ressortübergreifende Zusammenarbeit auch Wissenszuwächse oder der Transfer von Know-how möglich. Für Schulen bzw. in diesem Fall für Lehrkräfte könnte dies bedeuten detaillierteres, strukturierteres und fundiertes Wissen über Gesundheitsförderung oder die oben bereits genannten Themen Ernährung, Bewegung, Stressregulation etc. zu generieren. Entsprechende Prozesse, die womöglich zu Veränderungen in der Unterrichtsgestaltung (z. B. in Form von gezielten Bewegungselementen im Sinne einer „bewegten Schule“) führen können, beschreiben Synergieeffekte und damit einen weiteren Mehrwert von Netzwerken. Über entsprechende Kooperationen, vielleicht sogar im Unterricht, kann zudem die Transparenz in Bezug auf Angebote oder Akteurinnen und Akteure in der Kommune (z. B. Sportvereine) erhöht werden und verschiedene Institutionen können sich gegenseitig Einblicke in ihr (berufliches) Handeln geben. Durch entsprechende Einblicke können wiederum gezielt Doppelstrukturen entweder entwickelt oder abgebaut werden. Ein Beispiel dafür wäre die gemeinsame Nutzung von Sportanlagen durch Schulen und Sportvereine oder gemeinsame Schwimmkurse. Anhand dieser möglichen Form der Zusammenarbeit lässt sich gut illustrieren, dass der effektivere Einsatz bzw. die effektivere Nutzung von Ressourcen ein weiterer Mehrwert von Netzwerken sein kann. Durch diese könnten Sportvereine möglicherweise auch Zugangshürden abbauen und neue Teilnehmerinnen und Teilnehmer für ihre Sportangebote finden. Die systematische Vernetzung der verschiedenen relevanten Settings innerhalb einer Kommune oder eines Stadtteils wird „Supersetting Approach“ [17] genannt und betont die Vorteile des Zusammenwirkens der unterschiedlichen Institutionen und Disziplinen [15]. Durch den interdisziplinären Austausch professioneller Perspektiven können Problemlagen effektiver sowie effizienter gelöst werden. Beispiele hierfür wären möglicherweise die Bewältigung von psychosozialen Problemen von Schülerinnen und Schülern durch Kooperation mit einer Jugendberatungsstelle oder, im Falle von auffälligen oder schwerwiegenderen psychischen Problemen, die Zusammenarbeit mit einer psychiatrischen oder psychotherapeutischen Ambulanz.

Anhand der von den Autorinnen und dem Autor aufgeführten Beispiele in Bezug auf die Mehrwerte von Netzwerken im Rahmen der Netzwerkarbeit von Schulen wird deutlich, wie vielfältig Netzwerkpartnerinnen und -partner sein können und welche – hier beispielhaften – Mehrwerte aus Sicht der Schule entstehen können. Gleichzeitig deuten einige Beispiele auch auf Mehrwerte für Netzwerkpartnerinnen und -partner. Dabei sind, wie deutlich wird, die Anzahl an Partnerinnen und -partnern wie auch die Zwecke der gemeinsamen Arbeit im wechselseitigen Interesse vielfältig. Zudem deuten die angesprochenen synergetischen Effekte darauf hin, dass diese parallel oder als aufeinanderfolgende „Kettenglieder“ (z. B. in Präventionsketten) eintreten können und somit keineswegs isoliert zu betrachten sind. Anknüpfend an die Darstellungen zu Eigenschaften und Nutzen von Netzwerken soll im folgenden Abschnitt der Frage nachgegangen werden, welche möglichen Zugänge Schulen mit Blick auf Netzwerkarbeit und in Bezug auf Netzwerkpartnerinnen und -partner haben bzw. wie sie unter dem gesundheitsförderlichen Blickwinkel vorgehen könnten.

## Netzwerkpartnerinnen und -partner – Perspektiven der Zusammenarbeit

Ein leistungsfähiger Indikator in Bezug auf die Frage, welche Partnerinnen und Partner Teil von schulischen Netzwerken zu Gesundheitsförderung sein könnten, sind die Daten von Schuleingangsuntersuchungen, wie zum Beispiel Groos und Kersting [18] sowie Groos und Jehles [19] eindrucksvoll nachweisen konnten. Die Autorinnen und Autoren konnten in ihren hier beispielhaft genannten Untersuchungen nicht nur zeigen, welchen Entwicklungsstand Kinder zum Zeitpunkt des Eintritts in die Regelbeschulung aufwiesen, sondern setzten diese in hierarchischen Modellen ins Verhältnis zu anderen kommunalen Mikrodaten (zum Beispiel der Anteil der Sozialgeldbeziehenden im Sozialraum oder ob die besuchte Kindertagesstätte in einem Brennpunkt lag). Hogrebe und Pomykaj ([20], vgl. a. [21]) nutzten ähnliche Zugänge, um die „KiTa-Komposition“^2^ im Verhältnis mit den Sprachkompetenzen der Kinder zu betrachten. Die oben genannten Untersuchungen aus dem Projekt „Kein Kind zurücklassen! Kommunen in NRW beugen vor“ (heute: Kommunale Präventionsketten) zeigen in mehrfacher Weise, wie wichtig Kenntnisse über lokale oder regionale Gesundheitsdaten bei der Auswahl von Netzwerkpartnerinnen und -partnern für Schulen bzw. für Kommunen sind. Auf diese Weise lassen sich einerseits mögliche Ursachen im Sinne von (sozialräumlichen) Risiko- und Schutzfaktoren ermitteln – was sicher größerer Bemühungen bedarf – wie sich andererseits Hinweise in Bezug auf den Gesundheitszustand der Schulanfängerinnen und -anfänger zeigen.^3^ So können anhand dieser Daten, wie oben beschrieben, Themen bzw. Bedarfe identifiziert werden, anhand derer Netzwerkpartnerinnen und -partner ausgewählt werden können.

An dieser Stelle zeigt sich deutlich die enge Assoziation von kommunaler und schulischer Gesundheitsförderung. Gleichzeitig wird die Rolle der Kommune als Netzwerkpartnerin bzw. Akteurin unterstrichen. Das heißt, dass Schule und Kommune im Sinne der bereits in der Einleitung genannten Weise voneinander profitieren und die Schule aufgrund ihrer sozialräumlichen Einbindung dann die Chance hat, zielgerichtete Netzwerkarbeit zu betreiben, wenn die Bedingungen im Sozialraum oder Stadtteil sowie gleichzeitig die Rolle im Zuge der kommunalen Gesundheitsförderungsstrategie klar sind. Damit ist in Bezug auf das Verhältnis zwischen Kommune und Schule zudem deutlich, welchen wechselseitigen Vorteil im Sinne eines Synergieeffekts (die Identifikation von Themen und Bedarfen) beide haben können.

Neben den (zum Beispiel durch die Vernetzung mit kommunalen Akteurinnen und Akteuren oder auch Hochschulen bzw. Wissenschaftlerinnen und Wissenschaftlern) auszumachenden Bedarfen hinsichtlich gesundheitsförderlicher Angebote im Setting, Sozialraum, Stadtteil oder der Kommune, die aus dem und im Netzwerk generiert werden können, spielen bei der Vernetzung von Schulen auch die Bedürfnisse der Schülerinnen und Schüler eine große Rolle. Die bedeutsame Unterscheidung von Bedarfen als „objektiv“ gegebene Anknüpfungspunkte in Form von vorhandenen Ressourcen oder Defiziten und individuellen Bedürfnissen konnten Quilling und Müller [22] und Quilling et al. [23] anhand des vom Landeszentrum Gesundheit NRW geförderten Projekts „Partizipative Gesundheitsförderung“ darlegen. Hier zeigte sich, dass die Perspektiven auf gesundheitsrelevante Aspekte oder Probleme an Schulen durch Lehrkräfte auf der einen Seite und Schülerinnen und Schüler auf der anderen Seite sehr unterschiedlich eingeschätzt werden. Zur Ermittlung entsprechender Bedürfnisse spielt die Beziehung zwischen den Lehrkräften und den Schülerinnen und Schülern ebenso eine bedeutsame Rolle wie die Beziehung bzw. die Netzwerke unter den Schülerinnen und Schülern (Binnennetzwerke)^4^.

Entsprechend ist die Schülerinnen- und Schülerschaft im Sinne der lebensweltlichen Interpretation ihrer Umwelt ein ebenso wichtiger Ausgangspunkt für die Identifikation von relevanten Netzwerkpartnerinnen und -partnern. An dieser Stelle zeigt sich wiederum deutlich, wieso in Bezug auf Netzwerkarbeit ein breiter Begriff von Lebenswelt hilfreich ist (vgl. oben).

Auf dieser Basis können Netzwerkpartnerinnen und -partner unter anderem Sportvereine, Jugendhilfe, (Jugend‑)Beratungsstellen, Gesundheitsämter, andere Schulen, Ärztinnen und Ärzte (im Sozialraum oder Stadtteil), Psychotherapeutinnen und -therapeuten, Abenteuerspielplätze oder Landwirtinnen und -wirte sein. Beispiele für erfolgreiche gesundheitsförderliche Netzwerke und gleichzeitig Beispiele für die Vielfalt von gesundheitsförderlichen Netzwerken für und mit Schulen sind in Tab. 1 zusammengefasst.

**Table 1:** 

Netzwerk/Programm	Onlinepräsenz	Region
Gesund aufwachsen im Saarland	https://www.das-saarland-lebt-gesund.de/	Saarland
Schulen für Gesundheit 21	–	Gesamte Bundesrepublik Deutschland
Gesund leben lernen	https://www.gesundheit-nds.de/index.php/arbeitsschwerpunkte-lvg/bildungseinrichtungen/2-gesund-leben-lernen	Niedersachsen
Landesprogramm Bildung und Gesundheit NRW	https://www.bug-nrw.de/	Nordrhein-Westfalen
Modellprojekt Schulgesundheitsfachkräfte	https://schulgesundheitsfachkraft.de/	Brandenburg, Hessen
Pakt für Prävention Hamburg	https://www.hag-gesundheit.de/arbeitsfelder/pakt-fuer-praevention	Hamburg
Tutmirgut – gesunde Schule	https://www.rhein-sieg-kreis.de/gesundheit-soziales/gesundheit/gesundes-aufwachsen/tutmirgut.php	Nordrhein-Westfalen
Vernetzungsstelle Kita- und Schulverpflegung NRW	https://www.kita-schulverpflegung.nrw/	Nordrhein-Westfalen
Partnerprozess „Gesundheit für alle“	https://www.gesundheitliche-chancengleichheit.de/partnerprozess/	Bayern, Hessen
Gute gesunde Schule	https://www.ggs.bayern.de/	Bayern
Schools for Health	https://www.schoolsforhealth.org/	Europa
International School Health Network	http://www.internationalschoolhealth.org/	International
UNESCO Chair Global Health & Education	https://unescochair-ghe.org/	International

*Kita* Kindertagesstätte, *UNESCO* engl. United Nations Educational, Scientific and Cultural Organization, dt. Organisation der Vereinten Nationen für Bildung, Wissenschaft und Kultur

## Förderliche Faktoren und Hürden

Bedingungen der Zusammenarbeit von verschiedenen Institutionen im Bereich der Gesundheitsförderung sowie Aspekte des Gelingens und Scheiterns waren in den letzten Jahren Gegenstand verschiedener Arbeiten. Im Bereich schulischer Gesundheitsförderung wurden hierbei sowohl Bedarfe und Konsequenzen von Vernetzung auf Ebene der Akteurinnen und Akteure in der Schule als auch entsprechende förderliche Faktoren auf Ebene der institutionellen Zusammenarbeit untersucht.

Samdal und Rowling [26] konnten im Rahmen einer Synthese aus internationalen Studien zeigen, dass zur Realisierung von gesundheitsförderlichen Schulen neben Partnerschaften und Vernetzung als unabhängigem Faktor auf politischer Ebene vor allem die (gesundheitsförderliche) Planung der Schulentwicklung sowie deren politische und institutionelle Verortung bedeutsam sind. Mit Blick auf die Bundesrepublik Deutschland lassen sich hier neben dem Bundesministerium für Gesundheit vor allem die Bundeszentrale für gesundheitliche Aufklärung, die kommunalen Gesundheitsämter (Öffentlicher Gesundheitsdienst), die Kultusministerkonferenz sowie die Schulministerien nennen.

Auf institutioneller Ebene ließen sich die Form der Führung und des Managements ausmachen. Auf der operativen Ebene spielen die Ausbildung des Personals und die Beteiligung der Schülerinnen und Schüler eine große Rolle [26]. Alle genannten Aspekte müssen nachhaltig institutionalisiert und organisiert sein. Anknüpfend an den vorangehenden Punkt kam Ekornes [27], die Lehrkräfte zu interprofessioneller Zusammenarbeit im Kontext der Förderung psychischer Gesundheit befragte, ebenfalls zu dem Ergebnis, dass neben Zeitmangel (Ressourcen), kontextbezogenem Verständnis und der Schulleitung auch die selbst eingeschätzte Kompetenz ein wichtiger Faktor zu sein scheint. Darüber hinaus wurde aber auch hier deutlich, dass fehlende Vernetzung auch auf der individuellen Ebene subjektiv als Hemmnis wahrgenommen wird, die Lehrkräfte sich aber gleichzeitig als Gatekeeper sehen, was die mehrschichtige Bedeutung von Netzwerkarbeit an Schulen im Kontext von Gesundheitsförderung unterstreicht.

Weist et al. [28], die für das School Mental Health Program in den USA eine entsprechende Untersuchung hinsichtlich hemmender und förderlicher Faktoren in Bezug auf Netzwerkarbeit anstellten, konnten zeigen, dass eine fehlende Identifikation mit dem Programm bzw. eine Bagatellisierung der Ziele, nicht funktionierende interdisziplinäre Teamarbeit, mangelnde Koordination, mangelndes Vertrauen sowie Ressourcenprobleme zu den größten Herausforderungen gehörten. Mehrere Untersuchungen von Pucher et al. [29–31] in Bezug auf Gelingensfaktoren für nachhaltige Zusammenarbeit im Kontext von Gesundheitsförderung an Gesamtschulen zeigten, dass neben den oben genannten Faktoren die des sogenannten DISC-Modells (Diagnosis of Sustainable Collaboration) eine große Rolle zu spielen scheinen. Dazu gehören Aspekte der Unterstützung bzw. Förderung von Zusammenarbeit sowie deren Wahrnehmung, die Absichten und Maßnahmen, zudem das Veränderungsmanagement (Changemanagement), Projektmanagement, der Kontext, externe Faktoren und Nachhaltigkeit.

In den genannten empirischen Betrachtungen konnten die Autorinnen und Autoren zeigen, dass die Förderung der oben genannten Aspekte, zum Beispiel die Entwicklung einer gemeinsamen Vision oder die Etablierung klarer und funktionierender (Management‑)Strukturen (zum Beispiel eine Netzwerkkoordination), ein förderlicher Kontext sowie eine positive Einstellung der Akteurinnen und Akteure (zum Beispiel durch vorausgehende positive Erfahrungen), zu Verbesserungen etwa bei der Konsensbildung oder beim Eingehen von Verpflichtungen, aber auch beim Angleich von Strategien führte. Gleichzeitig führten enge bürokratische Strukturen und zu lang andauernde Verhandlungen dazu, dass positive Entwicklungen verhindert wurden.

Vor diesem Hintergrund ist festzustellen, dass Netzwerkarbeit Ressourcen kostet und zusätzlicher Arbeit bedarf, bevor Synergieeffekte genutzt werden und zu Erleichterung führen können. Zudem lässt sich konstatieren, dass es mindestens eine Person an der Schule geben muss, die sich für das Netzwerk verantwortlich fühlt und gleichzeitig die Fähigkeit besitzt, zwischen vielen verschiedenen Fachkräften zu moderieren und diese zu motivieren.

Netzwerke scheitern, wenn keine gemeinsamen Ziele definiert werden. Sie fungieren als zentraler Anker für die Motivation und das Engagement im Netzwerk. Konkrete Meilensteine helfen dabei, die Zwischenziele zu evaluieren und aus der Zielerreichung neue Motivation zum Durchhalten zu gewinnen. Darüber hinaus können konkurrierende Interessen der Netzwerkarbeit im Wege stehen. Daher gilt es, neben den gemeinsamen Interessen auch die Partikularinteressen transparent zu machen, um Enttäuschungen vorzubeugen. Es muss immer wieder Aushandlungsprozesse geben, bei denen die Entscheidungsfindung transparent stattfinden und die Länge der Aushandlung möglichst kurz gehalten werden sollte. Wenn die Netzwerkpartnerinnen und -partner das Gefühl haben, nicht adäquat beteiligt zu sein, werden sie ihr Engagement zurückfahren und möglicherweise aus dem Netzwerk austreten. Zu den Hindernissen erfolgreicher Netzwerkarbeit zählen auch eine unklare Rollenverteilung und intransparente Kommunikationsstrukturen.

## Diskussion und Fazit

Die vorliegende Arbeit konnte aufzeigen, weshalb Netzwerkarbeit aus verschiedenen Perspektiven eine zentrale Aufgabe von Schulen für die erfolgreiche Umsetzung von Gesundheitsförderung darstellt, die sich jedoch nicht zwangsläufig aus Lehrplänen ergibt und daher als Zusatzaufgabe aus Sicht der Lehrkräfte wahrgenommen wird. Die schulische Lebenswelt nimmt eine immer größer werdende Bedeutung im Alltag von Kindern und Jugendlichen ein, da sie einen wesentlichen Teil ihrer Zeit dort verbringen. Insofern ist es notwendig, Schule nicht als Ort der Wissensvermittlung zu verstehen, sondern als Lebenswelt, die gesundes Aufwachsen, Lernen und Arbeiten ermöglichen muss. Mithilfe systematischer Netzwerkarbeit kann das Setting Schule mit und für alle Beteiligten zu einer gesundheitsfördernden Lebenswelt transformiert werden. Es konnte dargestellt werden, welche Partnerinnen und -partner und welche Hinweise Schulen nutzen können, um Kooperation bedarfsorientiert aufzubauen. Gleichzeitig wurde gezeigt, dass neben Bedarfen, die aus und durch Netzwerke generiert werden, diese gleichzeitig dadurch kompensiert bzw. Lösungsperspektiven erarbeitet werden können, bei denen auch die Bedürfnisse der Schülerinnen und Schüler eine wichtige Rolle spielen. Hierbei ist der Kontakt der Lehrkräfte zu den Schülerinnen und Schülern bedeutsam (Binnennetzwerke).

Am Ende bleibt die Hoffnung, dass die Relevanz von Netzwerken bei der Umsetzung der Ziele von WHO und Schule weiterhin anerkannt wird und dass Netzwerke weiter genutzt und systematisch ausgebaut werden.

## Footnotes

1 Grundlegende Verfügungen zur Gesundheitsförderung: z. B. § 2 Abs. 6 Nr. 2 u. 8 Schulgesetz Nordrhein-Westfalen (SchulG NRW), § 6 SchulG HE (Hessen), § 1 Abs. 5 Nr. 2 Sächs(isches) SchulG, § 4 Abs. 5 Nr. 13 Brandenburgisches (Bbg)SchulG [5–7].

2 Das Konstrukt „KiTa-Komposition“ definierten die Autorinnen aus den Anteilen an Kindern aus Armut, mit Migrationshintergrund, mit Sprachförderbedarf, ohne nonformale Bildungsangebote, mit unvollständigen Vorsorgeuntersuchungen und mit Frühgeburt.

3 Auf kommunaler Ebene ergeben sich hier auch ohne umfangreicheres Matching von Daten, wie es im Falle der Analyse von Zusammenhängen zu anderen kommunalen Mikrodaten der Fall wäre, bereits Möglichkeiten der Analyse sozialräumlicher Unterschiede.

4 Eine umfangreiche Auseinandersetzung zu entsprechenden Netzwerken und ihren Folgen findet sich bei Bicer et al. [24]. Siehe dazu auch [25].
